# Plasticity of the fatty acid whole blood lipidome in the progression of canine periodontal disease: a pilot study

**DOI:** 10.3389/fvets.2025.1644675

**Published:** 2025-12-18

**Authors:** Carolina Silva, Tiago Sousa, Marisa Pinho, Ana Carolina Fontes, Ana Carolina Abrantes, Hugo Brancal, Francisco Peixoto, Rosário Domingues, Carlos Viegas

**Affiliations:** 1 Department of Veterinary Sciences, School of Agricultural and Veterinary Sciences (ECAV), University of Trás-os-Montes e Alto Douro (UTAD), Quinta de Prados, Vila Real, Portugal; 2 Animal and Veterinary Research Center (CECAV)—AL4AnimalS, University of Trás-os-Montes e Alto Douro (UTAD), Vila Real, Portugal; 3 Veterinary Hospital of Covilhã, Quinta das Ferreiras, Covilhã, Portugal; 4 Mass Spectrometry Centre, LAQV-REQUIMTE, Department of Chemistry, University of Aveiro, Campus Universitário de Santiago, Aveiro, Portugal; 5 CESAM, Departament of Chemistry, University of Aveiro, Campus Universitário de Santiago, Aveiro, Portugal; 6 Faculty of Health Sciences, University of Beira Interior, Covilhã, Portugal; 7 School of Agriculture, Polytechnic University of Castelo Branco (ESA-IPCB), Quinta da Senhora de Mércules, Castelo Branco, Portugal; 8 Chemistry Center of Vila Real, University of Trás-os-Montes e Alto Douro (UTAD), Quinta de Prados, Vila Real, Portugal; 9 RISE-Health: Health Research Network, Faculty of Medicine, University of Porto, Porto, Portugal; 10 CIVG—Vasco da Gama Research Center, University School Vasco da Gama (EUVG), Campus Universitário, Coimbra, Portugal

**Keywords:** dog, fatty acids, gingivitis, lipidomics, periodontal disease, periodontitis, whole blood

## Abstract

**Introduction:**

Periodontal disease is a progressive infectious-inflammatory disease, and, in the case of dogs, it is one of the most frequently diagnosed pathologies. Fatty acids (FA) play a dual role in inflammatory processes, as they have anti-inflammatory and pro-inflammatory functions. The goals of this study were to compare the FA profiles of whole blood in dierent degrees of canine periodontal disease in a single breed, the Portuguese Podengo, and consequently, to analyze the possible variation of these FA with the progression of the disease.

**Methods:**

Whole blood FA values were determined in healthy dogs, dogs with gingivitis and dogs with periodontitis by gas-chromatography-mass spectrometry. The study sample included 10 dogs considered clinically healthy, 10 dogs with gingivitis and 9 dogs with periodontitis.

**Results:**

Arachidonic acid and omega-3 docosapentaenoic acid were significantly higher in periodontitis cases compared to the control group, but curiously the arachidic acid was lower in the gingivitis group compared to the control group. Total saturated FA was significantly lower in the periodontitis group compared to the control group, while the total polyunsaturated FA was significantly higher in gingivitis and periodontitis groups than in the control group. Omega-6 polyunsaturated FA was significantly higher in cases of periodontitis than in healthy dogs.

**Discussion:**

The results presented suggest that the systemic impact of canine periodontal disease is partly reflected in the lipidomic profile of whole blood FA. The potential roles of the FA identified have important implications for a better understanding of the pathogenesis of canine periodontal disease, being equally endowed with potential as biomarkers for diagnosis and disease progression.

## 1 Introduction

Periodontal disease (PD) is a progressive infectious-inflammatory disease resulting of an interaction between the host, the microbial biofilm of dental plaque and environmental factors (1, 2). Its staging is based on clinical observation of gingivitis and periodontitis, and can be classified based on the severity of clinical signs and lesions in four stages: PD 1 (gingivitis), PD 2 (early periodontitis with less than 25 % of attachment loss or at most), PD 3 (moderate periodontitis corresponding to 25–50 % of attachment loss), and PD 4 (advanced periodontitis characterized by more than 50% of attachment loss), according the American Veterinary Dental College (3). Gingivitis, the first and reversible phase of the disease, corresponds to inflammation confined to the gingival tissue. As the clinical picture worsens, gingival redness and swelling may cause ulceration and spontaneous bleeding (4). Periodontitis, on the other hand, is a chronic and irreversible inflammation of the periodontal tissues, namely the gums, cementum, periodontal ligament, and alveolar bone. This stage can lead to tooth mobility, tooth loss, bone resorption and osteomyelitis of the maxilla and/or mandible (4–6). There is a progressive nature between the two phases when it is not diagnosed and/or treated in a timely and effective manner (4–21).

The systemic impact of PD is real and widely recognized in human and veterinary medicine (6, 22). Given the significant systemic impact of this disease, Tamura et al. (23) developed a method for estimating periodontal pocket surface area, which has shown potential for assessing the effects of periodontitis on systemic conditions. Research into indices such as this or potential biomarkers is crucial for determining more accurately the relationship between canine PD and systemic inflammation. It is believed that this disease affects almost half of the world's human population (24, 25). In the case of dogs, canine PD is also one of the most prevalent pathologies in small animal clinics (4, 7, 26, 27), which is why it is of great interest. (1, 6).

Lipids are an essential class of molecules that are present in organisms. Its main biological functions are the constitution of cell membranes, and energy storage. Furthermore, they are essential as signaling molecules (28).

In the specific case of fatty acids (FA), their influence on cellular inflammatory responses is recognized), with the inflammatory process resulting from their incorporation into cell membrane phospholipids (29–33).

Furthermore, FA are considered important nutritional factors, and they have been implicated in the pathogenesis of periodontal disease (PD) (34). Different FA have been correlated to pro-inflammatory and anti-inflammatory properties, appearing to have a dual role in inflammation (33), with special emphasis on omega-3 (n-3) and omega-6 (n-6) FA (12, 17, 35–66).

In the case of PD, the interest of polyunsaturated FA (PUFA) is notable in studies carried out in both human (17, 25, 34, 55, 57, 67) and veterinary medicine (12). Specifically, n-3 PUFA have been associated with antibacterial, anti-inflammatory and inflammatory bone loss regulating effects, which justify their research interest in regulating periodontitis (25, 68–70), in addition to their beneficial and preponderant clinical role in pathologies with a marked inflammatory component (12), not only in the initial phase but also during resolution (12, 71, 72). Retecka et al. (23, 73) developed a study in which treatment with n-3 PUFA in PD promoted significantly lower rates of bleeding on probing, as well as other advantages (25, 74). Furthermore, EPA and DHA inhibit potential periodontal pathogens, such as *Porphyromonas gingivalis* (12, 68, 75). On the other hand, in the specific case of canine PD, a study by Lourenço et al. (12) showed that EPA and DHA supplementation had no benefits in terms of gingival score or disease progression. Therefore, these different results promote an additional need for research in this area. As far as the AA is concerned, this FA is involved in the process that results in bone loss in experimental periodontitis (76, 77). Data like this demonstrates the potential of FA as biomarkers of canine PD, not only in terms of diagnosis, but also in terms of disease progression and treatment. The FA can be analyzed through their profile, which is an integral part of the analysis of the individual's lipidome, which is increasingly the subject of research interest in veterinary medicine (32, 33, 73, 76–78), and will certainly be one of the components of personalized veterinary medicine in the future.

To the best of our knowledge, no study has been published about the profiles of FA in different stages of canine PD, especially in a single breed. Therefore, the aims of this study were to compare the FA profiles in whole blood, determined by gas-chromatography-mass spectrometry (GC-MS), in healthy dogs, dogs with gingivitis and dogs with periodontitis and, consequently, to analyze how the progression of the disease may affect the FA present, all of this based on their potential as biomarkers. This will, therefore, be the first approach to lipidomics in canine PD.

Among other possibilities, whole blood is one of the samples in which the FA profile can be investigated to determine the structural and functional molecular components and their respective quantity. The reason we chose to analyze the whole blood in this study is to simplify the analysis of this sample, especially since this is a first approach to this disease. Thus, there was no need to go through the centrifugation stage, after which we would still have to collect the plasma (79). In addition, this type of sample allows for the analysis of lipid variations at the cellular level (79).

## 2 Material and methods

### 2.1 Data collection

This study was approved by the Ethics Committee of University of Trás-os-Montes e Alto Douro (Ref. Doc74-CE-UTAD-2024) and was developed in cooperation with Veterinary Hospital of Covilhã, University of Aveiro and University of Trás-os-Montes e Alto Douro. The study sample comprised 29 medium-sized Portuguese Podengo dogs, which were included in three different groups, depending on the degree or absence of PD, i.e., healthy, with gingivitis or with periodontitis. The owner of each of the animals included in the sample was asked to sign an informed consent form authorizing the use of their animal's data in this study. Furthermore, given the characteristics of this investigation, the ARRIVE guidelines (80) were considered and followed.

For clinical reasons unrelated to this investigation, the blood samples were collected by puncturing the jugular veins and placed in tubes containing heparin anticoagulants and ethylenediamine tetra-acetic acid (EDTA). After that, they were processed promptly. Every animal was required to fast for 12 h. In the first instance, a complete blood count and a serum biochemical profile were carried out on each of the animals in order to detect any analytical alterations that would make it impossible to include them in this study. Therefore, only animals with no analytical alterations, as well as no detectable alterations on clinical examination, were considered for the sample in question. These analyzes were performed using automatic analyzers (BC-5000 Vet, Mindray Fujifilm Portugal, S.A., Vila Nova de Gaia, Portugal and Dri-Chem NX500V, Fujifilm Portugal, S.A., Vila Nova de Gaia, Portugal). The excess sample was frozen at −80 °C for 2 months, after which it was properly processed for FA analysis. Whole blood FA analysis was performed at the Chemistry Research Center of the University of Trás-os-Montes and Alto Douro and Mass Spectrometry Center at the Chemistry Department of the University of Aveiro by GC-MS.

### 2.2 Inclusion criteria

Based on their PD condition—healthy, gingivitis, or periodontitis—the animals were divided up into three groups. All the individuals had to be vaccinated, dewormed, and have a normal general clinical examination. They also had to be free of any evident analytical abnormalities that would indicate an endocrine, hepatic, infectious, neoplastic or renal disease.

### 2.3 Exclusion criteria

Exclusion criteria included being under 1 year old, having a systemic disease, receiving medical treatment, and being a female in heat, pregnant, or lactating. In addition, we also considered the fact that no animal would be allowed to participate in this study if it had undergone periodontal therapy or surgery within the preceding 12 months. In addition, no supplementation of n-3 FA could have been given to these dogs during the same previous period of time.

### 2.4 The characterization of each of the three groups under study

Presence of gingivitis, plaque, and any obvious signs of gingival recession, furcation or root exposure, and missing or moving teeth were visually evaluated in conscious dogs during a clinical examination. After this assessment, each animal was assigned to one of the three groups under study.

The first group, known as the control group, included 10 dogs. None of them showed any signs of dental calculus or plaque. Gingival inflammation was not observed, and the height and architecture of the alveolar margin remained unchanged.

The gingivitis group consisted of 10 dogs, which showed clinical signs compatible with this stage of canine PD. The members of this group had superficial gingival inflammation associated with oedema and marginal erythema. They could additionally exhibit slight spontaneous bleeding or bleeding on touch. No loss of insertion was acceptable, and the alveolar margin's size and architecture had to be normal. It should be noted that a slight accumulation of dental calculus can be seen.

The last group in this study corresponds to dogs diagnosed with periodontitis, thus comprising the periodontitis group, with a total of 9 dogs. In this case, the animals in question had to show classic signs of periodontitis, namely gingival recession, furcation exposure, tooth mobility or even tooth loss, in addition to those already mentioned for the gingivitis group.

### 2.5 . Lipid extraction from the samples

The lipid extraction was performed using a double extraction procedure using a solvent combination of methanol, chloroform, and water in final proportions of 2:1/0.8 (V/V/V), based on methods previous described by Bligh et al. (81) with some modifications (82). In summary, 200 μL of the samples were transferred to a glass tube to which 750 μL of cold methanol (MeOH) was added. Each tube was vortexed for 20 s and then sonicated for 20 s on ice. Following this, 2.5 mL of cold chloroform was added, and the tubes were incubated for 30 min on ice under shaking (75 rpm) with a magnet every 15–30 min. After 30 min incubation, 625 μL of Milli-Q water was added to each tube, vortexed for 20 s, and a further 10 min incubation on ice at 75 rpm. Finally, the extracts were centrifuged at 2,000 rpm for 5 min, allowing the lipid-rich lower phase to be collected in a new glass tube. The extracts were then dried under a stream of nitrogen, and the lipid residue was stored at −20 °C until quantification.

### 2.6 . Fatty acid profile from whole blood lipidome analysis

Whole blood in EDTA as anticoagulant was used to analyze the FA content by GC-MS following transmethylation. Thirty microgram aliquots of the lipid extracts were transferred to glass tubes and dried under a nitrogen stream. The lipid films were then dissolved in 1 mL of *n*-hexane containing C19:0 as an internal standard (1 μg mL^−1^, CAS number 1731-94-8, Merck, Darmstadt, Germany).

Each tube was added 200 μL of a potassium hydroxide (KOH) 2 M solution in methanol and the mixture was vortexed for 2 min. Next, 2 mL of a saturated sodium chloride (NaCl) solution was added, and the mixture was centrifuged for 5 min at 626 × *g* to promote phase separation. Cholesterol in the upper (organic) phase was removed using a 10 mm silica column in a pipette tip with wool pre-conditioned with 5 mL of hexane. Methyl esters were added to the column and eluted with a hexane and diethyl ether (95:5, *v*/*v*, 3 mL) mixture, then completely dried under a nitrogen stream. Finally, fatty acid methyl esters (FAME) were dissolved in 100 μL of *n*-hexane, and 2 μL of the resulting solution was injected into an Agilent Technologies 8860 GC System (Santa Clara, CA, USA) equipped with a DB-FFAP column (30 m length, 0.32 mm internal diameter, and 0.25 μm film thickness, J&W Scientific, Folsom, CA, USA).

The gas chromatograph was connected to an Agilent 5977B Network Mass Selective Detector, operating with electron impact ionization at 70 eV, scanning the mass range of *m*/*z* 50–550 in a 1 s cycle in full scan mode. The oven temperature program started at 58 °C for 2 min and increased by 25 °C min^−1^ to 160 °C, by 2 °C min^−1^ to 210 °C, and by 30 °C min^−1^ to 250 °C, holding for 10 min. The injector and detector temperatures were set at 220 and 280 °C, respectively. Helium was used as the carrier gas at a flow rate of 1.4 mL min^−1^. FA were identified by comparing retention times to those of commercial FAME standards in the Supelco 37 Component FAME Mix (ref. 47885-U, Sigma-Aldrich, Darmstadt, Germany) and by MS-spectrum comparison with chemical databases (Wiley 275 library and AOCS lipid library).

The relative percentages of FA were calculated using the percent relative area method.

### 2.7 . Statistical analysis

Statistical analyzes were performed using Jamovi statistical software (version 2.3.28). The continuous variables were assessed for normality using the Shapiro-Wilk Test. The values of each variable under study were analyzed using one-way analysis of variance (ANOVA, for data with a normal distribution) or the Kruskal–Wallis test (for data with a non-normal distribution). Values compared using one-way ANOVA were subjected to the Tukey or Games-Howell (FA 15:0, FA 16:0, FA 18:1n-7, FA 18:2n-6, FA 20:1n-9, FA 20:3n-6, FA 20:4n-6, FA 20:5n-3, FA 22:5n-6, total PUFA and n-6 PUFA) *post hoc* test; values compared using the Kruskal-Wallis test were subjected to the Dwass-Steel-Critchlow-Fligner (FA 14:0, FA 16:1n-7, FA 16:1n-9, FA 18:0, FA 18:1n-9, FA 18:3n-3, FA 20:0, FA 20:2n-6, FA 22:5n-3, FA 22:6n-3, total of saturated fatty acids (SFA), total monounsaturated fatty acids (MUFA) and n-3 PUFA) *post hoc* test. Differences between the study groups in terms of gender, age and weight were analyzed using Fisher's exact test, the Kruskal Wallis test and the one-way ANOVA, respectively. A *p*-value < 0.05 was considered statistically significant

## 3 Results

### 3.1 Study population

Three groups were defined for analysis in this study. The control group (C group) included 10 dogs, none of which showed any signs of plaque or gingival inflammation. In addition, the height and architecture of the alveolar margin remained unchanged. In turn, the 10 dogs belonging to the gingivitis group (G group), as the name suggests, showed clinical signs compatible with this stage, i.e., superficial gingival inflammation associated with oedema and marginal erythema, with the possible presence of dental calculus. In these cases, there was no loss of attachment, and the size and architecture of the alveolar margin were normal. The 9 dogs in the periodontitis group (P group) showed signs of this stage such as gingival recession, furcation exposure, tooth mobility or even tooth loss.

The main characteristics of the study sample (*n* = 29) are shown in Table 1. It should be noted that none of the dogs included in the sample were neutered. With regard to gender, it was possible to see that there was no statistically significant association between this variable and the stage of canine PD (*p* = 0.893), i.e., based on the classification of each of the three groups in the sample analyzed. Statistical analysis showed that there were also no statistically significant differences between the groups in terms of age (*p* = 0.394) and weight (*p* = 0.089).

**Table 1:** General characterization of study sample (*n* = 29).

**Group**	**Number of female (%)**	**Number of male (%)**	**Median (range) age of dogs (years)**	**Median (range) weight of dogs (kg)**
Control	5 (50.00%)	5 (50.00%)	1.50 (1.00–3.50)	14.00 (13.30–15.00)
Gingivitis	4 (40.00%)	6 (60.00%)	3.00 (2.00–3.75)	12.00 (11.00–13.00)
Periodontitis	3 (33.33%)	6 (66.67%)	3.00 (2.00–6.00)	13.00 (13.00–15.00)

### 3.2 Fatty acid-profiling from whole blood

A total of 19 FA were identified and quantified in the whole blood of 29 dogs, belonging to three different groups, according to their degree of PD. The FA identified (Table 2) were myristic acid, pentadecanoic acid, palmitic acid, 7-hexadecenoicacid, palmitoleic acid, stearic acid, oleic acid, vaccenic acid, linoleic acid (LA), α-linolenic acid, arachidic acid, gondoic acid, eicosadienoic acid, dihomo-gamma-linolenic acid, AA, EPA, n-6 DPA, n-3 DPA and DHA. The relative quantification of FA obtained in the three different groups, as well as the respective statistical analyzes, are described in Table 2, where different letters represent statistically significant differences. The graphical representation of these same results can be found in the Supplementary material.

**Table 2:** Fatty acids profiling identifies by GC-MS after alkaline trimethylation and expressed as relative abundance % obtained in control, gingivitis and periodontitis groups.

**Fatty acid**	**C group**	**G group**	**P group**	***p*-value**
Myristic acid (FA 14:0)	0.24 ± 0.17^a^	0.28 ± 0.27 ^a^	0.18 ± 0.09 ^a^	0.68
Pentadecanoic acid (FA 15:0)	0.13 ± 0.05 ^a^	0.21 ± 0.09 ^a^	0.15 ± 0.06 ^a^	0.06
Palmitic acid (FA 16:0)	16.00 ± 2.09 ^a^	15.50 ± 1.94 ^a^	14.70 ± 1.79 ^a^	0.35
7-hexadecenoic acid (FA 16:1n-9)	0.17 ± 0.06 ^a^	0.29 ± 0.24 ^a^	0.29 ± 0.18 ^a^	0.22
Palmitoleic acid (FA 16:1n-7)	0.55 ± 0.46 ^a^	0.35 ± 0.15 ^a^	0.52 ± 0.39 ^a^	0.77
Stearic acid (FA 18:0)	38.40 ± 7.36 ^a^	34.90 ± 3.07 ^a^	32.60 ± 2.89 ^a^	0.10
Oleic acid (FA 18:1n-9)	13.10 ± 4.80 ^a^	10.50 ± 2.05 ^a^	12.20 ± 3.64 ^a^	0.51
Vaccenic (FA 18:1n-7)	2.08 ± 0.89 ^a^	1.95 ± 0.41 ^a^	2.29 ± 0.25 ^a^	0.12
Linoleic acid (LA) (FA 18:2n-6)	12.90 ± 3.10 ^a^	14.10 ± 2.04 ^a^	12.30 ± 2.10 ^a^	0.21
α-Linolenic acid (FA 18:3n-3)	0.05 ± 0.07 ^a^	0.12 ± 0.15 ^a^	0.05 ± 0.08 ^a^	0.41
Arachidic acid (FA 20:0)	0.10 ± 0.09 ^a^	0.02 ± 0.03 ^b^	0.07 ± 0.10 ^ab^	0.04
Gondoic acid (FA 20:1n-9)	0.25 ± 0.07 ^a^	0.21 ± 0.06 ^a^	0.27 ± 0.05 ^a^	0.11
Eicosadienoic acid (FA 20:2n-6)	0.14 ± 0.09 ^a^	0.14 ± 0.09 ^a^	0.08 ± 0.08 ^a^	0.21
Dihomo-gamma-linolenic acid (FA 20:3n-6)	1.02 ± 0.41 ^a^	1.13 ± 0.33 ^a^	1.14 ± 0.28 ^a^	0.77
Arachidonic acid (AA) (FA 20:4n-6)	13.20 ± 4.96 ^a^	17.50 ± 2.84 ^ab^	20.10 ± 3.36 ^b^	0.01
Eicosapentaenoic acid (EPA) (FA 20:5n-3)	0.091 ± 0.12 ^a^	0.58 ± 0.55 ^a^	0.33 ± 0.34 ^a^	0.02
n-6 Docosapentaenoic acid (FA 22:5n-6)	0.63 ± 0.30 ^a^	0.68 ± 0.20 ^a^	1.29 ± 0.68 ^a^	0.06
n-3 Docosapentaenoic acid (DPA) (FA 22:5n-3)	0.32 ± 0.28 ^a^	0.52 ± 0.33 ^a^b	0.68 ± 0.46 ^b^	0.03
Docosahexaenoic acid (DHA) (FA 22:6n-3)	0.66 ± 0.905 ^a^	1.09 ± 1.02 ^a^	0.88 ± 1.21 ^a^	0.63

Different letters mean statistically significant difference.

The most abundant FA was stearic acid (FA 18:0) followed by palmitic acid (FA 16:0). At the opposite end, the lowest abundant FA detected was α-linolenic acid (FA 18-3). To date, and to the best of the authors' knowledge, no study has been carried out with similar characteristics to this one, particularly in terms of the type of sample and with the same profile of FA as this one. The studies carried out so far on the same species are based on erythrocyte membrane lipidome (33, 77), in which a cluster of 10 FA were identified, so an objective comparison with the results presented here is partly unfeasible. Even so, as far as the control group is concerned, the individual values of most of the FA are in the same order of magnitude as those mentioned in the other articles also developed in the canine species but in the erythrocyte membrane (33, 77).

The statistical analysis revealed that are changes in the profile of some FA, namely arachidic acid, AA, EPA and n-3 DPA. Arachidic acid showed a significantly lower abundance in gingivitis group when compared to the control group. In contrast, AA was significantly higher in periodontitis group than in control group. Regarding of EPA, although a statistically significant value was obtained in the ANOVA test, after carrying out the Games Howell test it was possible to verify that there were no statistically significant differences between the three groups. In relation to the n-3 DPA, the abundance obtained in the periodontitis group was significantly higher than in the control group. The individual FA not mentioned above showed no statistically significant differences between the three groups.

The total FA contents of the FA groups, namely total SFA, total MUFA, total PUFA, n-3 PUFA and n-6 PUFA were calculated for the three different groups, as well as the statistical analysis, are summarized in Table 3, which shows some statistically significant differences. Total SFA was significantly lower in periodontitis group than in control group. Regarding the total PUFA, the abundance obtained in gingivitis and periodontitis groups is significantly higher than in control group. N-6 PUFA was significantly higher in the periodontitis group than in control group. The other total FA contents showed no statistically significant differences between the groups. As with the individual analysis of the FA profile, these results are also represented graphically in the Supplementary material.

**Table 3:** Total fatty acid contents in control, gingivitis and periodontitis groups.

**Variable**	**Control group**	**Gingivitis group**	**Periodontitis group**	***p*-value**
Total SFA	54.80 ± 7.83 ^a^	50.90 ± 2.59 ^ab^	47.60 ± 3.08 ^b^	0.01
Total MUFA	16.10 ± 5.30 ^a^	13.30 ± 2.65 ^a^	15.60 ± 4.13 ^a^	0.34
Total PUFA	29.00 ± 6.80 ^a^	35.80 ± 3.56 ^b^	36.80 ± 2.72 ^b^	0.02
n-3 PUFA	1.12 ± 1.27 ^a^	2.31 ± 1.75 ^a^	1.94 ± 1.83 ^a^	0.17
n-6 PUFA	27.90 ± 6.32 ^a^	33.50 ± 3.00 ^ab^	34.90 ± 2.24 ^b^	0.02

Saturated Fatty Acids (Total SFA) = %C14:0 + %C15:0 + % C16:0 + % C18:0 + %C20:0; Monounsaturated Fatty Acids (Total MUFA) = % C16:1n-7 + %C16:1n-9 + %C18:1n-7 + % C18:1n-9 + %C20:1n-9; Total Polyunsaturated Fatty Acids (PUFA) = %C18:3n-3 + %C20:5n-3 + %C22:6n-3 + %C22:5n-3 + %C18:2n-6 + %C20:2n-6 + %C20:3n-6 + %C20:4n-6 + %C22:5n-6; n-3 Polyunsaturated Fatty Acids = %C18:3n-3 + %C20:5n-3 + %C22:6n-3 + %C22:5n-3; n-6 Polyunsaturated Fatty Acids = %C18:2n-6 + %C20:2n-6 + %C20:3n-6 + %C20:4n-6 + %C22:5n-6. Different letters mean statistically significant difference.

## 4 Discussion

Previous research in PD evidenced on the interplay of FA and inflammatory responses in periodontitis (25, 34, 67, 79, 83–92). The first FA for which there were statistically significant differences between the groups was arachidic acid, which showed a significantly lower abundance in gingivitis group when compared to that obtained in control group (90, 93–96). This preliminary result deserves further research in order to understand in more detail the role of this FA in canine PD. Furthermore, given the origin of arachidic acid from dietary sources, it may be important to analyze the individual's diet in detail in future investigations, since the reason for these differences may not only be due to the PD but also to the dietary sources. However, given the breed selected for this study and its aptitude for hunting, it is impossible to collect concrete and objective dietary data, since we know that there may always be uncontrolled food intake on the part of the owners. Besides all this, for a first approach to the profile of FA in canine PD, analyzing the diet was not one of the main objectives of this study.

AA was significantly higher in periodontitis group than in control group. This significantly higher abundance in cases of canine periodontitis was similar to that obtained in different studies carried out in humans (97–99). The combination of these results from several studies implies a probable involvement of high levels of AA in the pathogenesis of periodontitis (97).

AA plays a preponderant role in the inflammatory process, as it is involved in modulating its intensity and duration (34, 55, 97, 100–103), and its metabolism is directly affected by inflammation and oxidative stress (34), both so characteristic of canine PD. In our case, the obtained result suggests the central role that AA plays in the course of an inflammatory process, as PD, and how notorious its elevation is in these cases (34, 55, 97, 104, 105). These facts suggest that AA may have potential as a diagnostic biomarker for canine PD, although more studies are needed to confirm this possibility.

However, it would be a somewhat reductive view to consider that the abundance of AA in periodontitis group, compared to control group, is due exclusively to the consequent release of its best-known inflammatory mediators. This is because AA also plays a role in regulating inflammation, independently of its metabolites, demonstrating protective effects in inflammatory diseases mediated by the immune system (86). Moreover, AA has the ability to destroy bacteria by compromising their cell membranes (86, 106, 107), and as is well known there is a component of bacterial proliferation in oral cavity of dogs with canine PD (108, 109). For all these reasons, the authors of this study also hypothesized that the results relating to AA could also be due to its regulatory capacity in inflammation and its protective effect in immune-mediated inflammatory pathologies, such as canine PD (33, 86, 110–115).

The last FA in which there were statistically significant differences between the groups was the n-3 DPA, with the n-3 DPA in periodontitis group being significantly higher than in control group (32, 116). N-3 DPA has a role in the resolution of inflammation because, like EPA and DHA, it is a substrate for the synthesis of specific pro-resolving mediators with biological activity, such as resolvins and protectins (32, 117, 118). Our result is in line with that described in the study conducted by Figueredo et al. (97), which also obtained significantly higher n-3 DPA abundance in patients with generalized chronic periodontitis. However, in this author's case, the comparative term were patients with gingivitis only (25, 32, 34, 97, 118).

Considering the groups of SFA, MUFA and PUFA, some differences were observed.

The total SFA were significantly lower in periodontitis group than in control group. The SFA induce inflammatory signaling by stimulating TLR 2 and 4 (33, 119). TLR recognize pathogen-associated molecular patterns (PAMP) and trigger innate immune responses to protect the host from invasive pathogens (119). However, one of the peculiarities of some TLR is that they can be activated by endogenous non-microbial molecules, such as SFA, which consequently induces inflammation (119). In the case of canine PD, periodontitis is the degree to which a strong inflammatory component characterizes it. Therefore, it is not to be expected that in the control group, this high total SFA abundance would be synonymous with a potential activation of TLR and, consequently, translate into the induction of inflammation. However, the mechanism by which SFA induce TLR 2 and 4 is not so clear and explicit (119). It is therefore important to explore this, together with the present result, in future studies (25, 33, 55, 120).

The total PUFA in the gingivitis group and the periodontitis group is significantly higher when compared to the control group. In addition, it is possible to denote a significantly higher abundance of n-6 PUFA in individuals with periodontitis, which consequently contributed to the total PUFA being significantly higher especially in individuals with periodontitis, in addition to cases of gingivitis, compared to those clinically healthy (34).

Therefore, the known pro-inflammatory effect of n-6 PUFA should be emphasized (25, 121–123). Sztolztener et al. (124) suggested that changes in AA levels could be useful as indicators of the irreversible progression of inflammation. In our case, the AA value was also significantly higher in periodontitis group compared to control group. Furthermore, in the specific case of PGE_2_ and LTB_4_, eicosanoids originating from the metabolism of AA and promoters of inflammation (25, 125), these lipid mediators are associated with bone resorption, collagen destruction, oedema, inflammatory cell recruitment and pain, all of which are characteristics of PD (12, 126). These facts allow us to justify the results obtained in this study, both in terms of AA and n-6 PUFA, indicating the chronic inflammatory nature of this phase of canine PD (4, 83, 127).

It is important to emphasize that if we investigate a breed other than the Portuguese Podengo, in this case, the results presented may be different. This fact has already been shown in Doberman Pinschers and Boxers, due to obtaining different plasma concentrations of n-3 FA (73). It is, therefore, essential that, in studies of this kind, the sample in question is limited to a single breed, and a sample of different breeds should be avoided.

## 5 Limitations

Firstly, the main limitation of this study was the small sample size, reflecting its exploratory and preliminary nature (78).

Another limitation concerns the lack of accurate information on the diet of the dogs enrolled, given their aptitude for hunting and the consequent impossibility of dietary control. As such, the composition of the diet—particularly the exact concentration of FA—was not assessed. The inclusion of dietary data in future studies will be crucial, especially with regard to n-3 FA intake. However, we confirmed that none of the dogs received supplements highly enriched with these FA.

The use of whole blood as an analytical sample can also be considered a limitation, as plasma remains the most commonly used matrix (79). Nevertheless, this choice allowed us to explore a less conventional but potentially informative approach. Future studies using plasma will be important to determine whether lipid changes also occur at the cellular level (79). In addition, the erythrocyte membrane—known to reflect systemic health, nutritional, and metabolic status (32, 33, 77)—represents another promising sample type. In addition to plasma and erythrocytes, saliva should also be considered, as it has significant potential for periodontal research (128).

Finally, the limited number of studies investigating FA profiles in periodontal disease restricts the breadth of comparison and discussion of our results. These limitations can be overcome by expanding the sample size and incorporating lipidomic analyzes into future research. We also plan to include multiple dog breeds and apply advanced analytical techniques, such as liquid chromatography-tandem mass spectrometry (LC-MS/MS), which will allow for the evaluation of more complex lipid species, including oxylipids, ceramides, and phospholipids (129).

## 6 Conclusions

The findings indicate that alterations in whole-blood FA profiles reflect systemic inflammation associated with the progression of PD in dogs. Significant changes in specific FA and FA classes suggest a close link between lipid metabolism and PD pathogenesis. These FA may serve as promising biomarkers for early diagnosis and monitoring of canine periodontal disease.

## Data Availability

The raw data supporting the conclusions of this article will be made available by the authors, without undue reservation.
