# Supplementary material for: Plasticity of the fatty acid whole blood lipidome in the progression of canine periodontal disease: a pilot study
Source: Front Vet Sci. 2025 Dec 18;12:1644675. doi: 10.3389/fvets.2025.1644675 (PMC12756932; doi:10.3389/fvets.2025.1644675)
Supplement: Supplementary file 1 [file Table_1.DOCX]

Supplementary Material

**Graphic 1.** Fatty acids profiling identifies by GC-MS after alkaline trimethylation and expressed as relative abundance % (mean and standard deviation) obtained in control, gingivitis and periodontitis groups.

**Graphic 2.** Total fatty acid contents (mean and standard deviation) obtained in control, gingivitis and periodontitis groups.


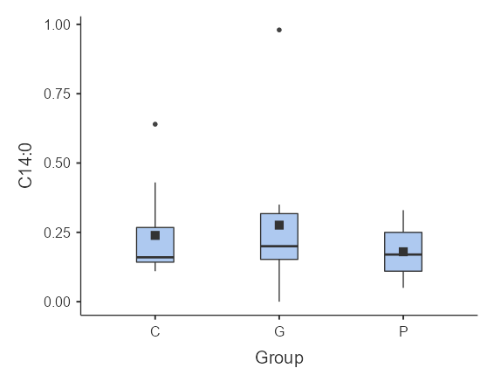

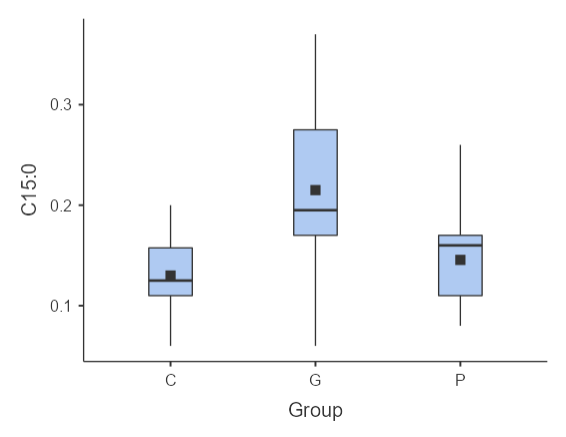


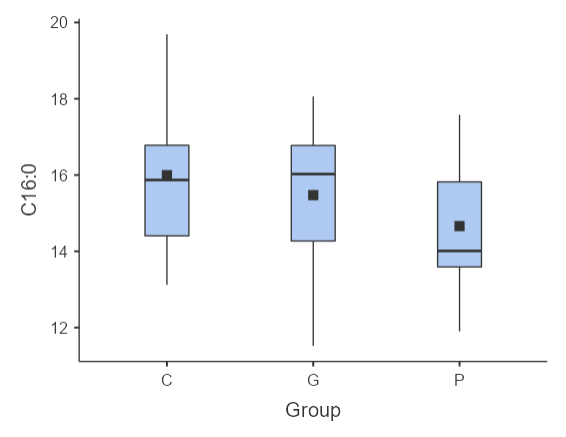

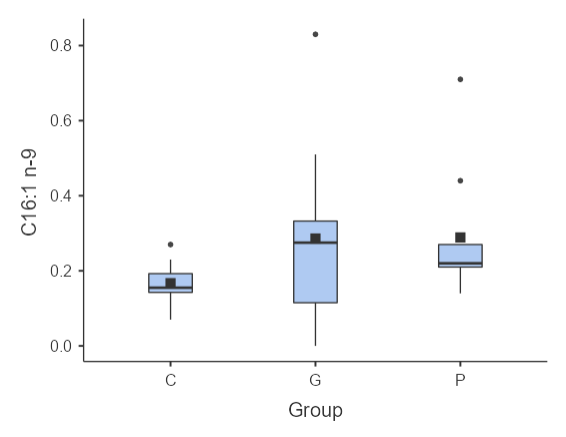


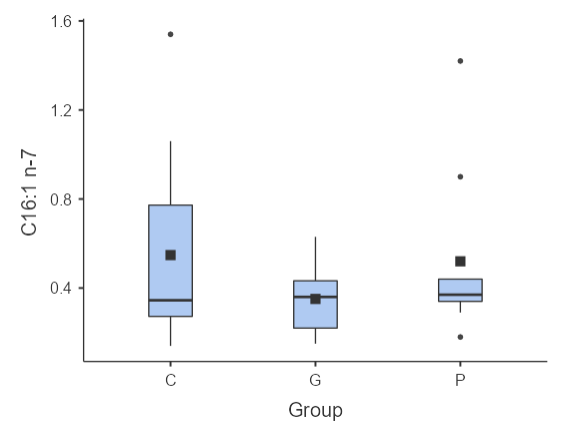

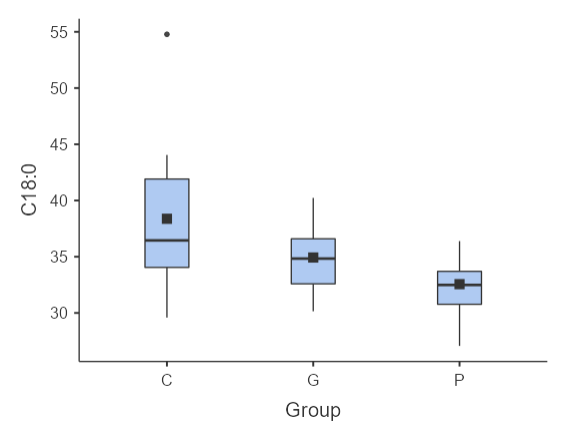


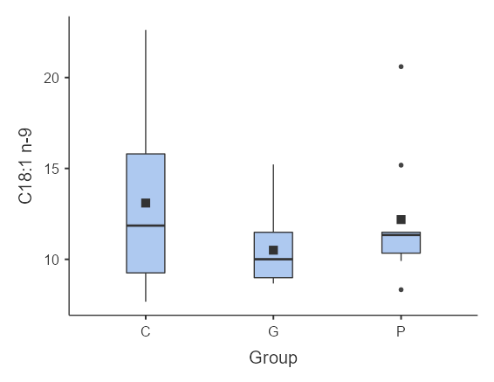

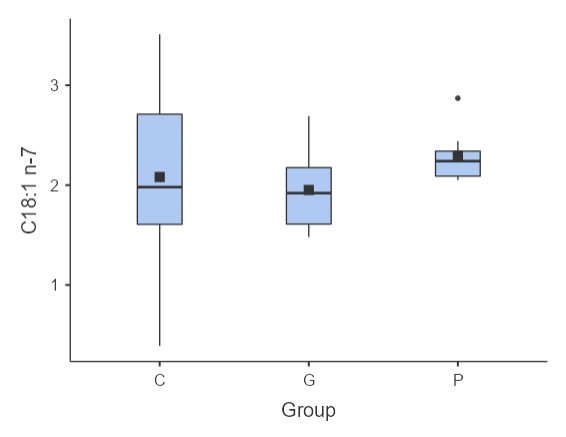


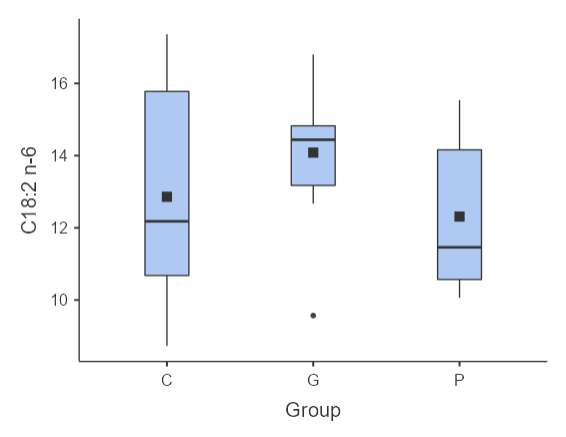

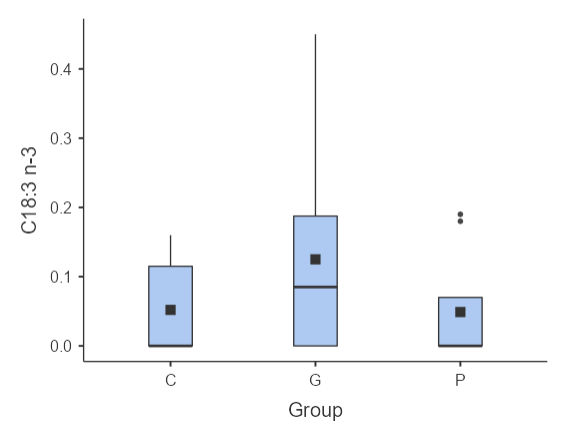


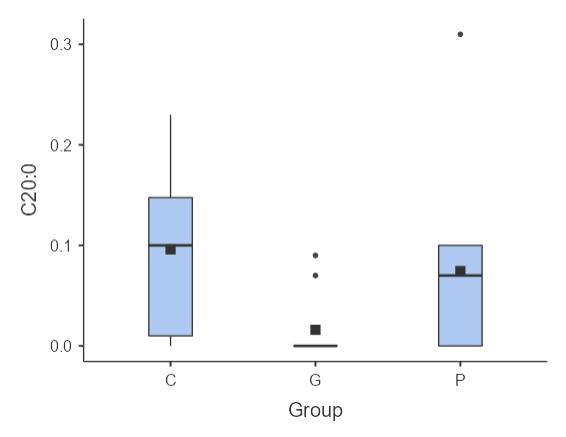

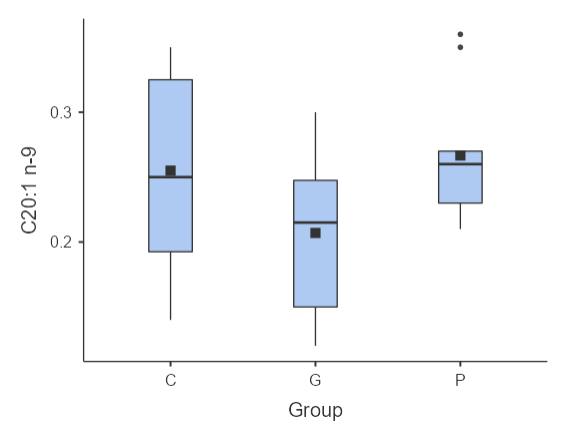


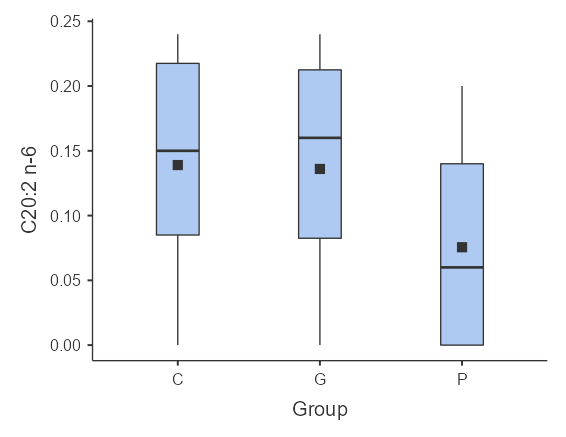

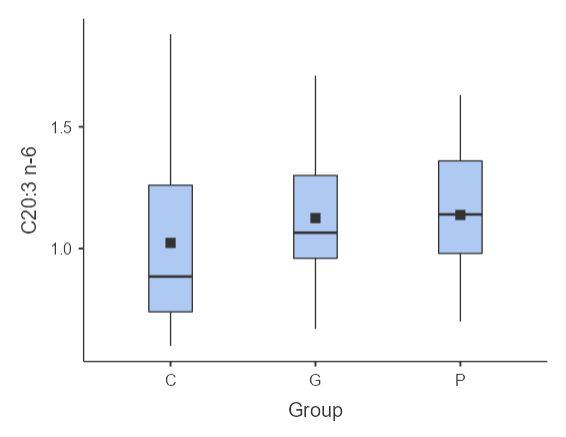


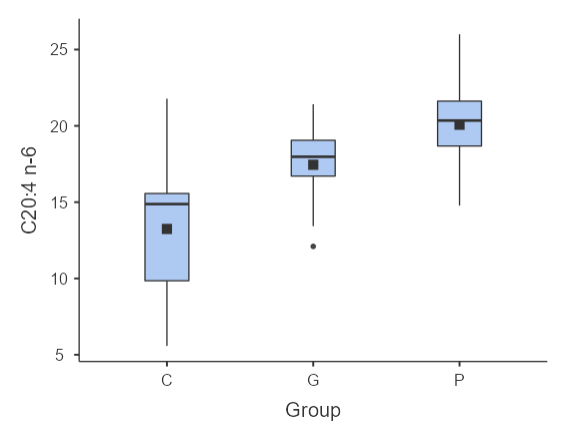

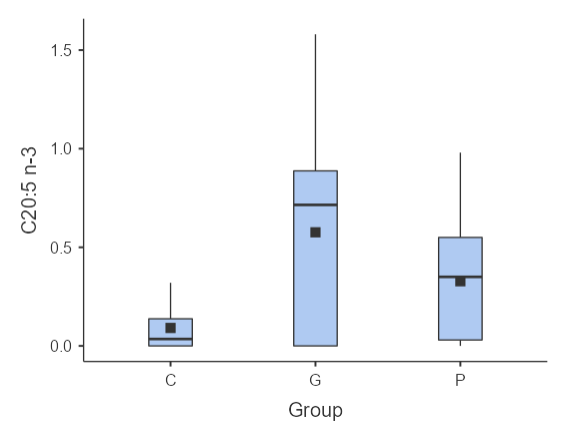


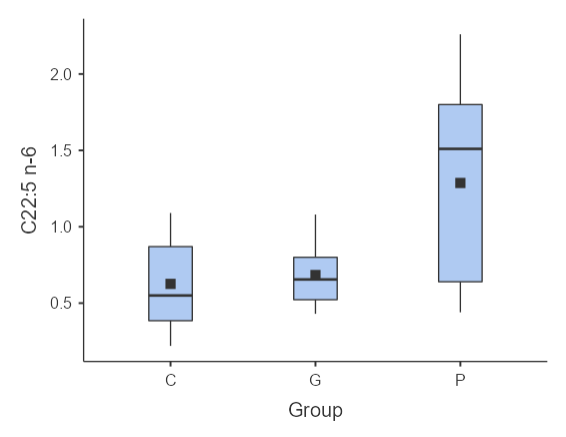

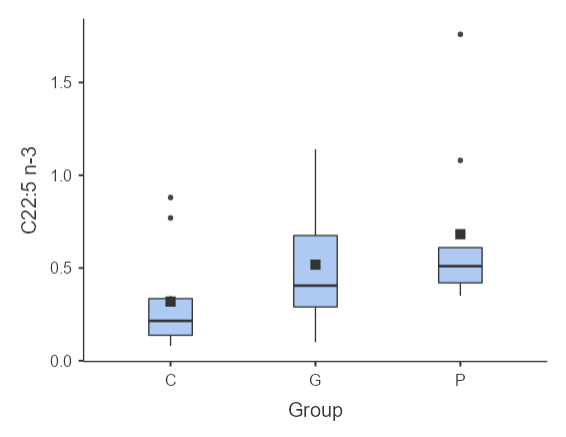


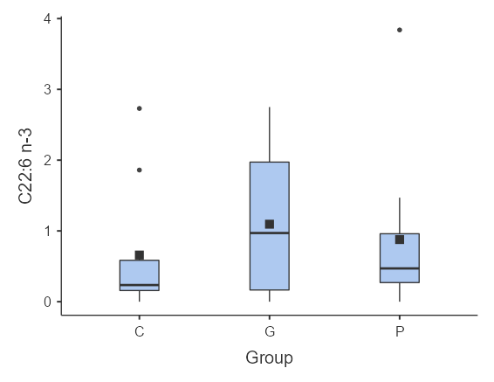


Figure 1. Box and whisker plot comparing the different fatty acids identified by GC-MS after alkaline trimethylation and expressed as relative abundance % (mean and standard deviation) obtained in control, gingivitis and periodontitis groups. The central square represents the mean value; the central horizontal lines indicate the median value; the upper and lower lines of the box plot represent the first and the third quartiles of the values, respectively. The outliers are represented with circles.


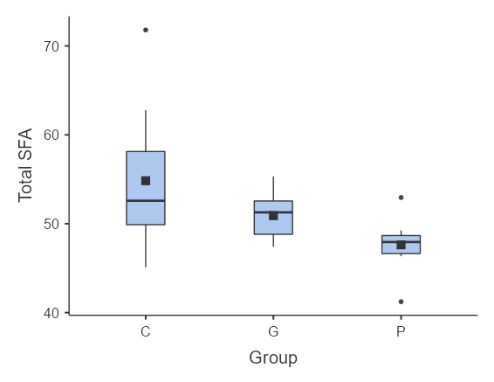

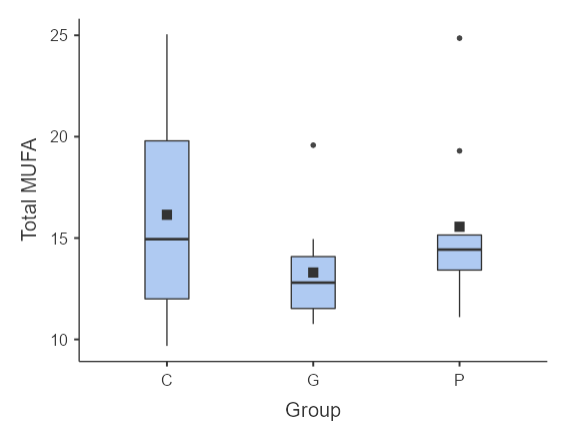


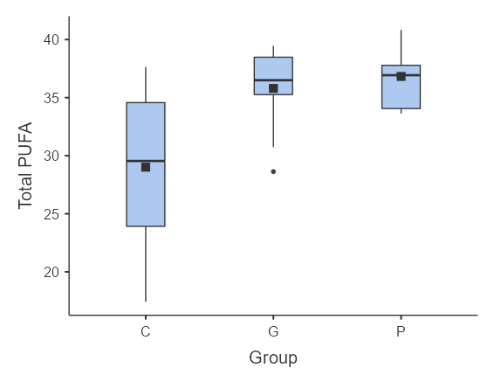

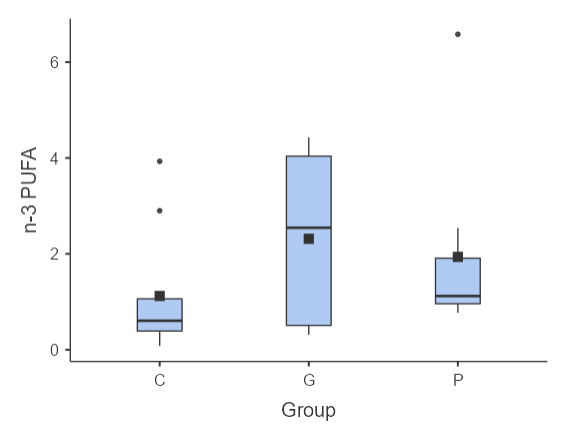


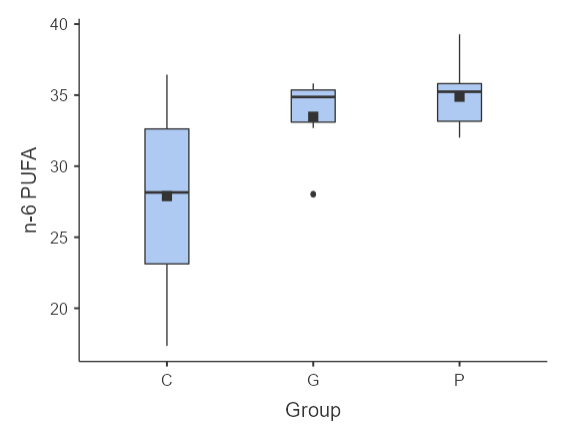


Figure 2. Box and whisker plot comparing the total fatty acid contents obtained in control, gingivitis and periodontitis groups. The central square represents the mean value; the central horizontal lines indicate the median value; the upper and lower lines of the box plot represent the first and the third quartiles of the values, respectively. The outliers are represented with circles.
